# Supplementary material for: Prevalence and risk factors of recurrent aphthous stomatitis among college students at Mangalore, India
Source: PeerJ. 2023 May 15;11:e14998. doi: 10.7717/peerj.14998 (PMC10194064; doi:10.7717/peerj.14998)
Supplement: Supplemental Information 3 [file peerj-11-14998-s003.pdf]

PREVALENCE AND RISK FACTORS OF RECURRENT APHTHOUS ULCERS- A CROSS SECTIONAL STUDY

matthewmanoj12@gmail.com (not shared) Switch account

* Required

Prevalence and Risk Factors associated with Recurrent Aphthous Ulcers among the age group of 18-30 years

Age *

Your answer

Sex *

Your answer

Course *

Your answer

Blood Group *

- A+
- A-
- B+
- B-
- AB+
- AB-
- O+
- O-

Have you been affected by mouth ulcers? *

- Yes
- No

Have you been affected by mouth ulcers in the past 6 months ? *

- Yes
- No
- Not Applicable

Does your family have a history of mouth ulcers? *

- Yes
- No

If you do get mouth ulcers do they occur in clusters or are they single mouth ulcers? *

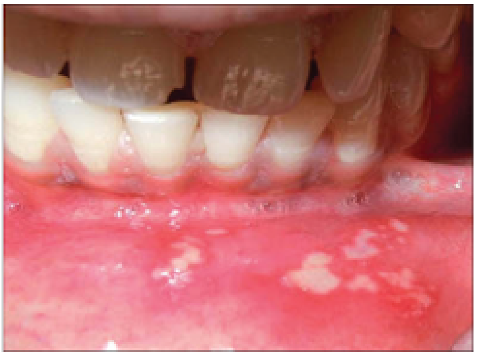

e 1: A cluster of aphthous ulcers

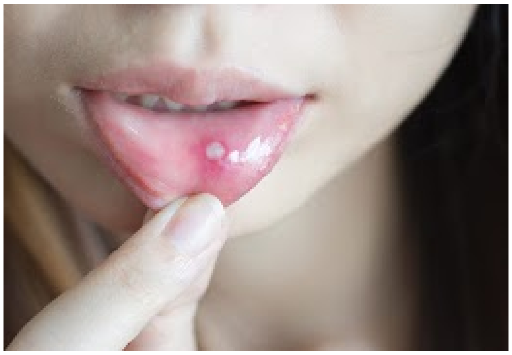

- Clusters
- Single Mouth Ulcers
- Both
- Not Applicable

Where do you usually get mouth ulcers? (Select as many as applicable) *

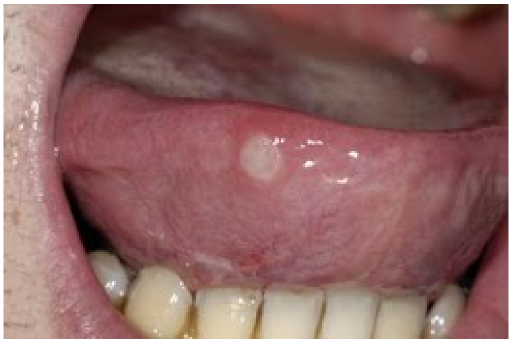

- [ ] On the surface of the tongue

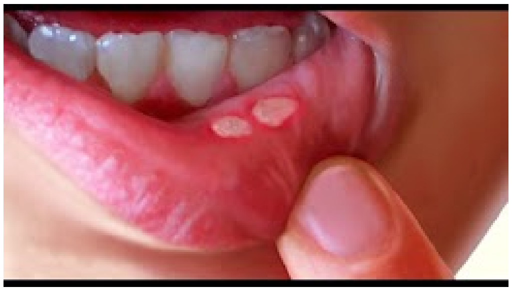

- [ ] Inside of the lips

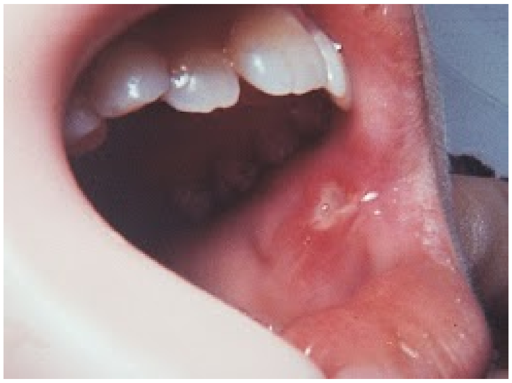

- [ ] Inner surface of the cheeks

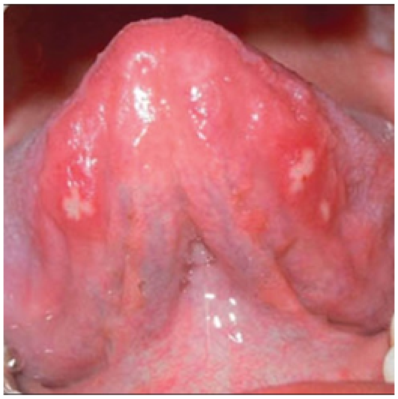

- [ ] Bottom of the tongue
- [ ] Not Applicable

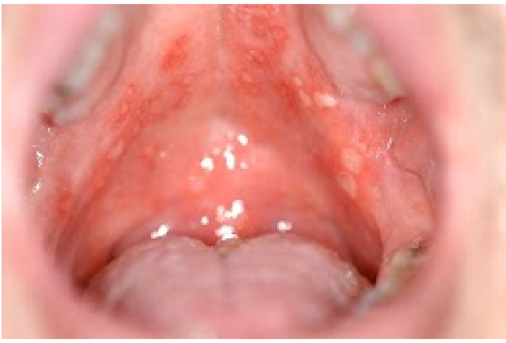

- [ ] Roof of the mouth

Are you a Diabetic patient? *

- Yes
- No

Does your family have a history of Diabetes? *

- Yes
- No

Do you smoke? *

- Yes
- No

If you stopped smoking, did you discover mouth ulcers during the initial stages when you stopped smoking? *

- Yes
- No
- Not Applicable

Do you get mouth ulcers because of biting your cheeks, biting your tongue, brushing vigorously, etc.? *

- Yes
- No
- Not Applicable

Have you ever worn braces/dentures? *

- Yes
- No

How many hours of sleep do you get regularly? *

- less than 5 hours
- 5-6 Hours
- 7-8 Hours
- More than 8 Hours

Do you use a toothpaste that foams on brushing? *

- Yes
- No

At what parts of the day do you brush your teeth? (Choose as many as applicable) *

- [ ] Morning
- [ ] Afternoon
- [ ] Evening
- [ ] Night
- [ ] After food
- [ ] Before food

For Female participants, have you been affected by ulcers specifically during menstruation? *

- ○ Yes
- ○ No
- ○ Not Applicable

Do you get mouth ulcers when you are stressed? *

- ○ Yes
- ○ No

Do you get mouth ulcers after the consumption of the following fruits? (Choose as many as applicable) *

- [ ] Pineapples
- [ ] Apples
- [ ] Figs
- [ ] Lemons
- [ ] Oranges
- [ ] Strawberries
- [ ] Bananas
- [ ] Not applicable

If there are any other particular fruit that gives you mouth ulcers, please mention it below.

Your answer

Does any of the following foods give you mouth ulcers? (Choose as many as applicable) *

- [ ] Chocolates
- [ ] Cheese
- [ ] Peanuts
- [ ] Almonds
- [ ] Eggs
- [ ] Spicy food
- [ ] Not applicable

If there are any other particular food that gives you mouth ulcers, please mention it below.

Your answer

Does any of the following beverages give you mouth ulcers? (Choose as many as applicable) *

- [ ] Coffee
- [ ] Tea
- [ ] Milk
- [ ] Carbonated Drinks
- [ ] Orange Juice
- [ ] Pineapple Juice
- [ ] Not Applicable

If there are any other particular beverages that gives you mouth ulcers, please mention it below.

Your answer

Tell us about your daily consumption of coffee? *

- ◯ Nil
- ◯ 1-2 Cups
- ◯ 3-4 Cups
- ◯ More than 5 cups

Tell us about your daily consumption of tea? *

- Nil
- 1-2 Cups
- 3-4 Cups
- More than 5 cups

Tell us about your daily consumption of Milk? *

- Nil
- 1-2 Cups
- 3-4 Cups
- More than 5 cups

How often do you drink carbonated drinks? *

- Daily
- 3-4 times a week
- 1-2 times a week
- Nil

Do you have any nutritional deficiencies, if yes please mention?

Your answer

Do you have any allergies? If so please mention?

Your answer

Do you suffer from any systemic diseases? *

- [ ] Crohn's Disease
- [ ] Coeliac disease
- [ ] Behçet’s disease
- [ ] Reactive arthritis
- [ ] Not applicable

Do you take any medication for your ulcer? (Choose as many as applicable) *

- [ ] Gel
- [ ] Tablets
- [ ] Vitamin supplements
- [ ] Mouth wash
- [ ] Home remedies
- [ ] Not applicable

Do you take any medication other than the above mentioned to treat your mouth ulcers, please mention it below.

Your answer

Back Submit Clear form

Never submit passwords through Google Forms.

This content is neither created nor endorsed by Google. Report Abuse - Terms of Service - Privacy Policy

Google Forms
